# Plasma amino acids imbalance in cirrhotic patients disturbs the tricarboxylic acid cycle of dendritic cell

**DOI:** 10.1038/srep03459

**Published:** 2013-12-10

**Authors:** Eiji Kakazu, Yasuteru Kondo, Takayuki Kogure, Masashi Ninomiya, Osamu Kimura, Yoshiyuki Ueno, Tooru Shimosegawa

**Affiliations:** 1 Division of Gastroenterology, Tohoku University Graduate School of Medicine, 1-1 Seiryo, Aobaku, Sendai, 980-8574 Japan; 2 Department of Community Medical Supports, Tohoku Medical Megabank Organization, Tohoku University, 2-1 Seiryo, Aobaku, Sendai, 980-8573 Japan; 3 Department of Gastroenterology, Faculty of Medicine, Yamagata University, 2-2-2 Iida-Nishi, Yamagata 990-9585, Japan; 4 CREST, Faculty of Medicine, Yamagata University, 2-2-2 Iida-Nishi, Yamagata 990-9585, Japan

## Abstract

An imbalance of plasma amino acids (AA) is observed cirrhotic patients. Here we report that the imbalance suppresses the maturation of dendritic cells (DCs) by reducing the intracellular ATP due to interference with the mitochondrial tricarboxylic acid (TCA) cycle. We used serum-free culture medium consistent with the average concentration of the plasma AA from a healthy volunteer (HCM) and that from patients with advanced cirrhosis (ACM). We compared the function of DCs and the metabolism of glucose-amino acids under each medium. The maturation and intracellular ATP of immature DCs were lower under ACM in spite of the enhancement of mitochondrial respiratory chain complex genes. Metabolomics revealed that the TCA cycle metabolite, fumarate and 2-oxoglutarate were increased in DCs generated under ACM. Consistent with *in vitro*, In CD1c^+^ or CD14^+^ cells from cirrhotic patients, the gene expression of 2-oxoglutarate-succinate-fumarate transition enzymes were significantly different from the cells of healthy controls.

Liver cirrhosis is the end stage of any type of chronic hepatitis^1^. Not only hepatocellular carcinoma but also bacterial infections, such as spontaneous bacterial peritonitis (SBP) or pneumonia, are frequent clinical complications in patients with advanced cirrhosis^2^. This means that patients with cirrhosis are immune-compromised hosts and has the immune abnormality. Several studies have reported the immunological abnormalities occurring in cirrhosis^3^,^4^, but it is not clear why the responses of immune cells are suppressed in patients with cirrhosis. On the other hand, in patients with advanced cirrhosis, various metabolic disorders involving glucose, protein-amino acids, lipids, vitamins, and minerals, appear, because liver is the most important organ for maintaining nutritional homeostasis. Regarding plasma amino acids, an imbalance with decreased levels of branched-chain amino acids (BCAAs) and increased levels of aromatic amino acids (AAAs), is commonly seen in patients with advanced cirrhosis^5^. Recently, it has become clear that amino acids are not only important as substrates for various metabolic pathways but also activate a nutrient-sensitive signaling pathway in synergy with insulin^6^,^7^. The mammalian target of rapamycin (mTOR) signaling pathway is one of the most representative pathways, and this pathway has been shown to act as a major effector of cell growth and proliferation through the regulation of protein synthesis^8^,^9^. Recent study reveals that malnutrition impairs interferon signaling through mTOR pathways in patients with chronic hepatitis C^10^, and we reported that the amino acids imbalance of patients with cirrhosis suppresses the maturation of DCs, accompanied by the down-regulation of the mTOR signal^3^,^11^. However, the mechanisms that underlie these phenomena are largely unknown. The mTOR senses cellular energy levels by monitoring the cellular ATP/AMP ratio via the AMP-activated protein kinase (AMPK)^12^. The phosphorylation of downstream effectors of mTOR is inhibited by rapamycin and activated by BCAA, especially by L-leucine^13^,^14^, although little is known about the impact of changes in the levels of extracellular amino acids on the immune system. In the immune system, especially DCs, toll-like receptors (TLRs) signaling is strongly modulated by mTOR^15^,^16^,^17^,^18^. On the other hand, TLR signaling promotes aerobic glycolysis and a decline in oxidative phosphorylation (OXHPOS), while glucose restriction prevents activation and leads to premature cell death^19^,^20^. The aim of this study, therefore, was to investigate the influence of the extracellular amino acid imbalance observed in patients with cirrhosis on the function of DCs and the energy metabolism.

## Results

### Amino acid concentrations similar to those in plasma of patients with advanced cirrhosis impaired the maturation of monocyte-derived dendritic cells (MoDCs)

**Figure 1:**
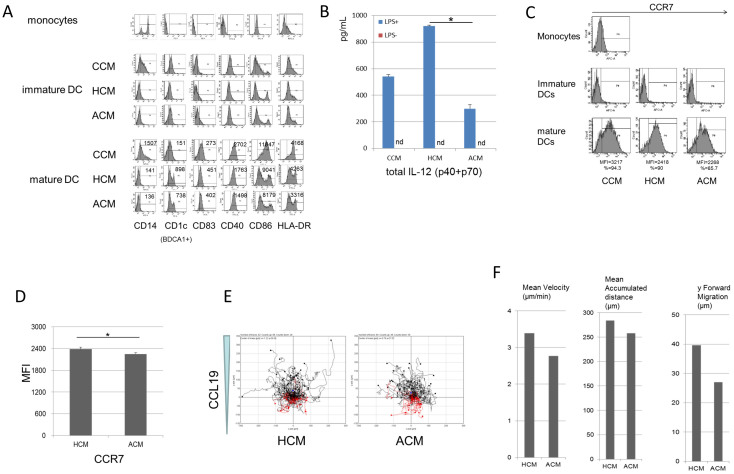
Amino acid concentrations similar to those in plasma of patients with advanced cirrhosis impaired the maturation of MoDCs. MoDCs from healthy volunteer were cultured from CD14+ cells under CCM, HCM and ACM in 6-well tissue culture plates for 10 days and exposed to 100 ng/mL LPS for an additional 24 h. (A) Cells were harvested before and after adding LPS, stained with different mAbs, and analyzed using flow cytometry. For the histogram figure, filled traces represent the marker-specific Ab; numbers indicate mean fluorescence intensity (MFI). Results are representative of five experiments from five different donors. (B) The supernatants were removed after adding LPS and assayed for the cytokine concentrations. Mean ± SEM values from three different donors are shown. (C) MoDCs were stained with APC-labeled anti-CCR7 before and after adding LPS. For the histogram figure, filled traces represent CCR7 Ab; percentages indicate positive cells. (D) MFI represented by Mean ± SEM values from five different donors are shown. (E) CCL19 chemotaxis of MoDCs after adding LPS. Results are representative of three experiments from three different donors. (F) Mean velocity, mean accumulated distance and y forward migration were measured by the chemotaxis and migration tool (IBIDI) for Image J. (B), (D)* p < 0.05.

First we generated MoDCs with GM-CSF and IL-4 under CCM, HCM and ACM, and evaluated the phenotypes of the MoDCs. The DC yield and viability were not different under any medium (data not shown). The CD14 + cells expressed CD14 bright, CD86 dim, and CD83 negative. Immature MoDCs expressed CD14, CD83 negative, and CD1c CD40 HLA-DR dim, but they did not express CD83. After adding LPS, mature MoDCs showed the up-regulation of costimulatory molecules (CD40 and CD86) and HLA-DR. These cells were also characterized by the induction of CD83 expression on their cell surface. We confirmed that these maturation markers of MoDCs were decreased under ACM compared to that under HCM (Figure 1A). Next, we measured IL-12, a cytokine that plays a pivotal role in the development of Th1-mediated cellular immune responses. The IL-12 production of MoDCs stimulated by LPS under ACM was significantly impaired (Figure 1B). In terms of the capacity of phagocytosis, there were no differences under each medium (Figure S1), (Movie S1). CCR7 is necessary to direct dendritic cells (DCs) to secondary lymphoid nodes^21^,^22^. Immature DCs were negative for CCR7 and, after adding LPS, the expression of CCR was up-regulated under each medium (Figure 1C). In agreement with other maturation molecules, the up-regulation of CCR7 was significantly suppressed under ACM compared to that under HCM (Figure 1D). Further, the chemotactic responses of MoDCs to the chemokine CCL19 concentration gradient were lower under ACM than under HCM (Figure 1E). Mean velocity, mean accumulated distance and y forward migration were decreased under ACM than under HCM (Figure 1F). These data indicated that the maturation of MoDCs was impaired under amino acid concentrations similar to those in the plasma of patients with advanced cirrhosis.

### Amino acid concentrations similar to those in plasma of patients with advanced cirrhosis reduced the intracellular ATP, accompanied by a low mitochondrial membrane potential

**Figure 2:**
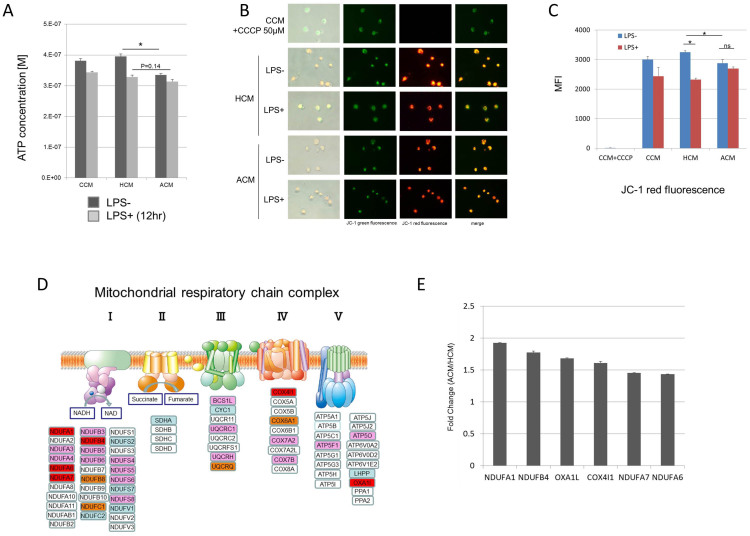
Amino acid concentrations similar to those in the plasma of patients with advanced cirrhosis reduced the intracellular ATP, accompanied by low mitochondrial membrane potential. MoDCs were generated from CD14+ cells under CCM for 10 days and re-cultured with or without 100 ng/mL LPS under CCM, HCM and ACM for an additional 24 h. (A) Intracellular ATP concentration was measured by luminometric assay. Mean ± SEM values from five different donors are shown. (B) Mitochondrial potential was assessed by fluorescence image with JC-1 dye. A representative image is shown. (C) JC-1 red fluorescence was determined by flow cytometry. Mean ± SEM values from three different donors are shown. (D) The expression of genes related to mitochondrial respiratory chain complex was determined by real-time PCR array. Out of 84, 71 genes could be determined. Red oval-genes are significantly higher (p < 0.05), orange oval-genes tend to be higher (0.05 < p < 0.10), and pink oval-genes are 1.4 fold higher under ACM than under HCM. Blue oval-genes are 1.4 fold lower under ACM than under HCM. (E) Six genes related to mitochondrial respiratory chain complex were significantly increased under ACM. Mean ± SEM values from three different donors are shown. (A), (C)* p < 0.05.

We investigated whether the amino acid imbalance in advanced cirrhosis influences the intracellular ATP level of MoDCs, because amino acid metabolism is closely related to the production of ATP in mitochondria. Luminometric assay revealed that the intracellular ATP level of MoDCs was significantly decreased in ACM before adding LPS. However, after adding LPS, there was no difference between HCM and ACM (Figure 2A). Regarding the mitochondrial activity, fluorescence microscope showed that, before adding LPS, the mitochondrial membrane potential was lower under ACM than that under HCM (Figure 2B). Interestingly, after adding LPS, the mitochondrial membrane potential was significantly decreased under HCM, but there was no difference under ACM after adding LPS (Figure 2B, 2C). Next, we determined the genes related to mitochondrial respiratory chain complex in ACM and HCM. Out of 84 genes, 71 could be determined. Out of 71genes, 26 were 1.4 fold higher under ACM than under HCM (Figure 2D). Some genes of complex I, IV, and V were significantly higher under ACM (Figure 2E). These data indicated that amino acid concentrations similar to those in plasma of patients with advanced cirrhosis reduced the intracellular ATP of MoDCs, accompanied by a low mitochondrial membrane potential in spite of the enhancement of mitochondrial respiratory chain complex genes.

### Amino acid concentrations similar to those in plasma of patients with advanced cirrhosis interfered with the TCA cycle by inducing an imbalance in the intracellular amino acids

**Figure 3:**
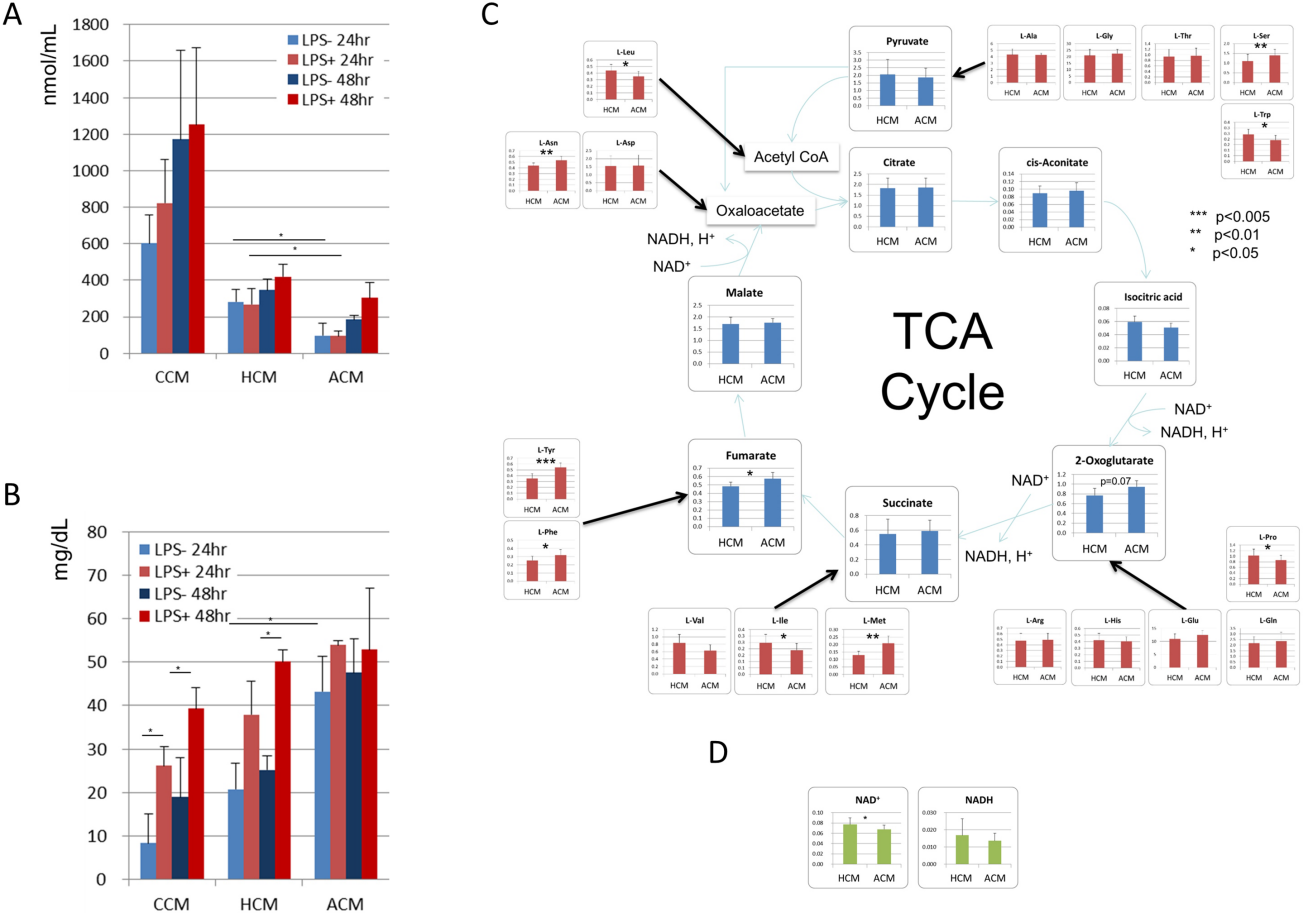
Amino acid concentrations similar to those in the plasma of patients with advanced cirrhosis interfered with the TCA cycle. (A) (B) MoDCs generated under CCM were re-cultured with or without LPS under CCM, HCM and ACM for an additional 48 h. The supernatants of each medium were measured by HPLC (for amino acids) and GOD methods (for glucose). (C), (D) MoDCs were cultured from CD14+ cells with GM-CSF and IL-4 under HCM and ACM for 7 days. Cells were analysed by CE-TOFMAS. The figure presents metabolites related to the TCA cycle. Value is relative area values/total detected values. (A), (B), (C), (D) *** p < 0.001, ** p < 0.01, * p < 0.05. Mean ± SEM values from three different donors are shown.

We investigated the amino acids and glucose consumption of MoDCs. Among 20 kinds of amino acids, every amino acid except L-Gly and L-Ala was decreased in both HCM and ACM (Figure S2A). The consumption of total amino acids except L-Gly and L-Ala was significantly lower under ACM than that under HCM regardless of adding LPS (Figure 3A). The consumption of L-Asp, L-Cys, L-Glu and BCAAs was higher with LPS than that without LPS (Figure S2B). On the other hand, the glucose consumption was at a low level before adding LPS under CCM and HCM, and the consumption was significantly increased after adding LPS (Figure 3B). Interestingly, under ACM the consumption was already at a high level and after adding LPS the consumption was not different (Figure 3B). The TCA cycle is a core pathway for the metabolism of amino acids^23^. Therefore, we hypothesized that the amino acid imbalance in the plasma of patients with advanced cirrhosis influenced the intracellular amino acids and the TCA cycle. To investigate this hypothesis, we performed metabolomics of MoDCs in ACM and HCM. The CE-TOFMS systems in two different modes for cation and anion analyses detected 163 peaks in MoDCs on average, and 159 of 163 metabolites could be analyzed. The difference in the relative levels of MoDCs metabolites between HCM and ACM were visualized by a using percentile heat map (Figure S3A). The heat map revealed that the concentration of the extracellular amino acids reflected to the concentration of the intracellular amino acids. L-leucine (L-Leu), L-isoleucine (Ile), L-tryptophan (L-Trp) and L-proline (L-Pro) were significantly decrease, and L-tyrosine (L-Tyr), L-phenylalanine (L-Phe), L-methionine (L-Met), L-serine (L-Ser) and L-asparagine (L-Asn) were significantly increased in ACM (Figure 3C). Among metabolites related to the TCA cycle, Fumarate was significantly increased and 2-oxoglutarate had a tendency to be increased in ACM (Figure 3C). Furthermore, nicotinamide adenine dinucleotide (NAD+), which is a coenzyme in the TCA cycle, was significantly decreased in ACM (Figure 3D). On the other hand, in the glycolytic pathway, the metabolites from glucose-6-phosphate (G-6-P) to glyceraldehyde-3-phosphate (G-3-P) had a tendency to decrease in ACM (Figure S3B). Regarding nucleotide metabolism, IMP was significantly decreased and ATP, ADP, GDP and GTP had a tendency to be decreased, while AMP was inversely increased in ACM (Figure S3C). These data indicate that the amino acid imbalance observed in the plasma of patients with advanced cirrhosis interferes with the TCA cycle mediators, especially 2-oxoglutarate-succinate-fumatate transition, and impair the nucleotide metabolism in MoDCs.

### Circulating DCs and monocytes from patients with advanced cirrhosis showed gene abnormalities in TCA cycle and the upstream regulators of mTOR

**Figure 4:**
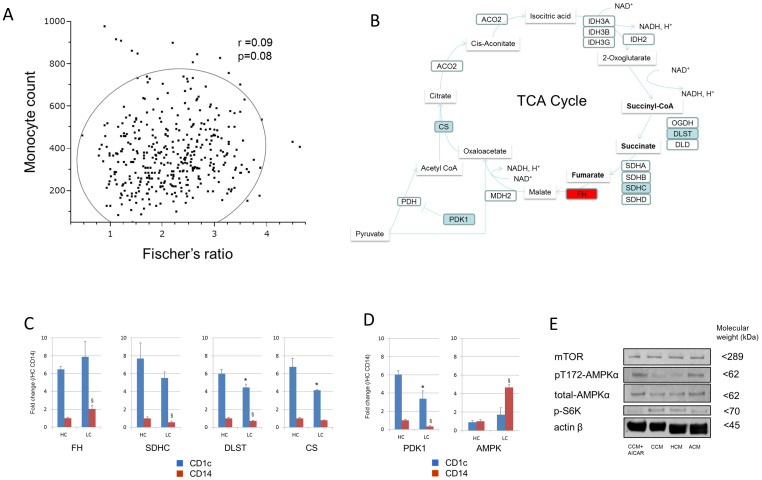
CD1c+ or CD14+ cells from patients with advanced cirrhosis showed gene abnormalities in TCA cycle and the upstream regulators of mTOR. (A) The correlation between monocyte count and Fischer's ratio. Fischer's ratio means: Valine + Leucine + Isoleucine/Tyrosine + Phenylalanine. Ellipsoids reflect the correlation envelopes for cut-off (0.95) in the total population. r; the coefficient of determination. (B) CD1c+ or CD14+ cells were isolated from healthy volunteers (n = 4) and patients with advanced cirrhosis (n = 8) (Table S2). The TCA cycle-related genes were determined by real time PCR. Red oval-gene is significantly higher (p < 0.05), and blue oval-genes are significantly lower in CD1c+ or CD14+ cells from patient with advance cirrhosis. (C) Relative expression of FH, SDHC, DLST and CS mRNA. (D) Relative expression of AMPK and PDK1 mRNA. (E) MoDCs were generated from CD14+ cells under CCM for 10 days and re-cultured under CCM, HCM and ACM for an additional 24 h. Cells were harvested and lysed. Equal amounts of protein were loaded and the levels of mTOR, phosho-p70 S6K, phosphor-T172 AMPK and total AMPK were determined by Western blot analysis. The blots and gels were cropped from full-length blots/gels presented in Figure S4. (B), (C) Mean ± SEM values from healthy controls (HC: n = 4) and patients with cirrhosis (LC: n = 8) are shown. *Value of P < 0.05 vs. CD1c of HC. §Value of P < 0.05 vs. CD14 of HC.

**Figure 5:**
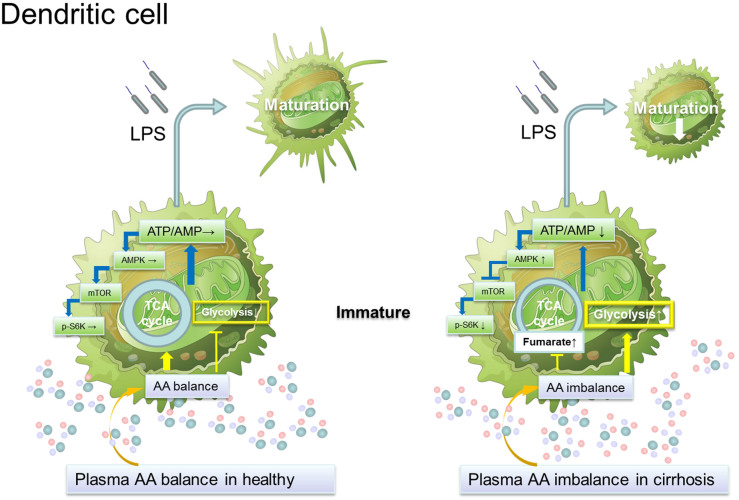
An imbalance of plasma amino acids in patients with advanced cirrhosis suppresses the maturation of dendritic cells by reducing the intracellular ATP due to interference with the mitochondrial TCA cycle. Immature DCs are sufficiently supplied with ATP by TCA cycle- OXPHOS in healthy volunteer. The change from the TCA cycle to glycolysis, which is needed for the maturation of DCs, is adequately operated under a healthy condition. However, in patient with cirrhosis, an imbalance of plasma amino acids decreased the intracellular ATP of immature DCs due to interference with the mitochondrial TCA cycle and OXPHOS, especially 2-oxoglutarate-succinate-fumarate transition. On the other hand, mTOR signaling was impaired in CD14+ cells from patients with cirrhosis consistent with the high expression of AMPK.

As in the in vivo study, we measured the concentrations of plasma amino acids of 107 chronic hepatitis and 270 cirrhotic patients by HPLC. We confirmed the imbalance of various amino acids in the plasma of patients with advanced cirrhosis (Table S1). There was no correlation between monocyte count and Fischer's ratio (Figure 4A). We selected 8 patients (Table S2) with advanced cirrhosis for analyzing the genes of CD1c+ or CD14+ cells involved in the regulation and enzymatic pathways of the TCA cycle (Figure 4B). Consistent with the metabolomics, fumarate hydratase (FH) mRNA was higher, and succinate dehydrogenase complex, subunit C (SDHC), dihydrolipoamide S-succinyltransferase (DLST) and citrate synthase (CS) were lower than in healthy controls (HC) (Figure 4C). Furthermore, the upstream regulators of mTOR, AMPK mRNA were higher and PDK1 mRNA was lower than in HC (Figure 4D). Finally, we investigated whether the amino acid imbalance observed in the plasma of patients with advanced cirrhosis influenced these nutrient related signalings. As expected, phosphorylation of AMPK was higher and phosphorylation of S6K was lower in MoDCs cultured under ACM than those under HCM or CCM (Figure 4E). These data revealed that the gene expression of TCA cycle enzymes, especially enzymes of 2-oxoglutarate-succinate-fumarate transition, was actually abnormal in CD1c and CD14 cells from patients with advanced cirrhosis. Consistent with the suppression of mTOR signal in vitro, CD1c+ or CD14+ cells showed gene abnormalities in the upstream regulators of mTOR. We provide a schematic diagram of the present study concerning abnormalities in the DCs of patients with advanced cirrhosis (Figure 5).

## Discussion

Previously, our study revealed that the plasma amino acids imbalance of advanced cirrhosis suppresses the maturation of dendritic cells (DCs) and that BCAAs partially improved the cytokine production^11^. However, the mechanisms that underlie these phenomena are largely unknown. In this study, we used two serum-free media (HCM and ACM) to be more representative of the human physiological environment. We could analyze the effect of each amino acid in the condition of cirrhosis only through the use of these media. First, we confirmed that the amino acid imbalance in the plasma of patients with advanced cirrhosis impaired the maturation of MoDCs. Regarding the process of DC maturation, there are four steps: i) antigen uptake, ii) antigen presentation, iii) migration to lymphoid organs, iv) interaction with T cells^24^. In this study, it was revealed that the imbalance impaired antigen presentation (CD40, CD80, CD86, HLA-DR) and, especially, the migration to lymphoid organs (CCR7) after adding LPS. Actually, the CCL19 chomotaxis of MoDCs was decreased under ACM. A previous study revealed that intracellular ATP of DCs was decreased after phagocytosis^25^, and described that DCs consume more ATP after the addition of a stimulating antigen. In Agreement with these reports, the luminometric assay revealed that the intracellular ATP of MoDCs was significantly decreased after adding LPS under HCM, but the intracellular ATP of MoDCs under ACM was not different after adding LPS. These results indicate that the intracellular ATP was consumed in order to mature under a healthy amino acid environment, but such consumption did not occurr under the amino acids environment of cirrhosis because the MoDCs already lacked ATP and the maturation of MoDCs was impaired. Reflecting ATP starvation, many genes related to mitochondrial respiratory chain complex were significantly higher under ACM. Interestingly, the genes of complex II, which are related to the oxidation of succinate to fumarate, were not different. Glucogenic amino acids are closely related to ATP production and give rise to the production of pyruvate or TCA cycle intermediates^26^. Accordingly, we investigated whether the amino acid imbalance of ACM influences the intracellular TCA cycle of immature MoDCs. As expected, CE-TOFMS revealed that some intracellular amino acids and TCA cycle intermediates, especially fumarate, were significantly increased under ACM, accompanied by low ATP and NAD+ under ACM. These data suggest that the increase in fumarate under ACM may constitute a negative-feedback mechanism for lowering the genes of complex II. On the other hand, the glucose consumption of MoDCs was significantly increased by LPS under HCM, but under ACM it was not changed by LPS because the consumption was already significantly higher without LPS. These data indicate that the amino acid imbalance of cirrhosis promotes aerobic glycolysis in immature DCs. Previous reports found that TLR signaling promotes aerobic glycolysis and a decrease in TCA cycle and oxidative phosphorylation (OXHPOS)^19^,^20^. We think that after adding LPS, the energy metabolism of DCs cannot shift from the TCA cycle to glycolysis under the amino acid imbalance of cirrhosis because glycolysis was already promoted in DCs before adding LPS. As an important limitation, we could not reveal in detail whether these amino acid changes occurred in the cytoplasm or mitochondria, because the fractionating procedure of mitochondria from the cytoplasm greatly affects the intracellular amino acids. Furthermore, we need to investigate autophagy, which is induced by amino acid starvation^27^ and influences the immune responses^28^. These issues should be evaluated in future studies.

Second, we investigated which amino acids are more strongly related to the maturation of DCs. Previously, we found that BCAAs, especially L-valine and L-leucine, increased the BDCA1+DCs allostimulatory capacity and IL-12 production under the amino acid environment of cirrhosis^11^. However, the enhancement by a single amino acid was very subtle and the effect of other amino acids was unknown. In this study, it was revealed that, except for BCAAs, L-aspartate, L-cystine and L-glutamate were more consumed for the maturation of DCs. L-aspartate and L-glutamate are very important for energy metabolism, because these amino acids are closely related to the malate-aspartate shuttle, which is a biochemical system for translocating electrons produced during glycolysis^29^. Furthermore, previous studies reported that L-cystine/L-glutamate antiport via xCT influences the intracellular GSH/GSSG ratio and cytokine production in monocytes and macrophages^30^,^31^,^32^. In agreement with these reports, the intracellular GSH of MoDCs was more decreased under ACM than under HCM in this study (Figure S3A). The administration of these amino acids may improve the function of DCs in patient with advanced cirrhosis. Concerning the molecular mechanism, it is known that a lack of intracellular ATP activates the phosphorylation of AMPK^33^ and that the activation of AMPK signaling suppresses mTOR signaling^34^. In agreement with these reports, the phosphorylation of AMPK was increased and the phosphorylation of S6K was decreased in MoDCs cultured under ACM.

Finally, we investigated the expression of genes related to the TCA cycle and the upstream regulators of mTOR in circulating DCs and monocytes from patients with cirrhosis. Consistent with in the vitro data, fumarate hydratase (FH) mRNA was higher, and succinate dehydrogenase complex, subunit C (SDHC), dihydrolipoamide S-succinyltransferase (DLST) and citrate synthase (CS) were lower than in healthy controls. Furthermore, the impaired mTOR signaling under ACM was consistent with the high expression of AMPK and the low expression of PDK1 in DCs and monocytes from patients with advanced cirrhosis.

In summary, we have shown that the plasma amino acid imbalance that appears in patients with cirrhosis impaired the maturation of DCs because the imbalance decreased the intracellular ATP of immature DCs due to interference with the mitochondrial TCA cycle and oxidative phosphorylation (OXPHOS), especially 2-oxoglutarate-succinate-fumarate transition. The change from the TCA cycle to glycolysis, which is needed for the maturation of DCs and is observed under a healthy condition, is impaired under the cirrhotic condition. The data from this study provide a rationale for future studies utilizing nutrition therapies, which could improve the immune function of patients with advanced cirrhosis.

## Methods

### Patients and healthy volunteers

The concentrations of plasma amino acids from 388 chronic hepatitis or cirrhotic patients were measured by high performance liquid chromatography (HPLC) in the early morning. Briefly, sulfosalicylic acid was added to plasma to a final concentration of 5%. The samples were then placed on ice for 15 minutes followed by centrifugation to remove precipitated proteins. The extracts were then analyzed for the amino acid content with a JLC-500/V (Ja-pan Electron Optics Laboratories, Tokyo, Japan). Also, these patients were classified according to the Child-Pugh classification. The monocyte was measured by a Beckman Coulter LH 750 Analyzer (Fullerton, CA, USA). CD14+ or CD1c+ cells were obtained from eight patients with advanced cirrhosis and four healthy volunteers. Written informed consent was obtained from each individual and the study protocol was approved by the Ethics Committee of Tohoku University School of Medicine (2009-209, 2010-323).

### MoDCs generation

PBMC were separated from the peripheral blood of healthy volunteers by centrifugation on a density gradient (Ficoll-Paque Plus, Amersham Biosciences). The CD14+ cells were isolated from PBMC using magnetic microbeads (Miltenyi Biotec, Bergish Gladbach, FRG). Cells were cultured with 1,000 U/mL GM-CSF and 500 U/mL IL-4 (PEPROTECH EC, London, UK) at a density of 1.0 × 10^6^ cells/well in 6-well flat-bottom plates for 10 days in a serum-free culture medium consistent with the average concentration of plasma amino acids from healthy volunteers, defined as the healthy control medium (HCM); whereas that from patients with advanced cirrhosis was defined as the advanced cirrhotic medium (ACM). The control culture medium containing all twenty kinds of amino acids was defined as the complete culture medium (CCM) (Table S3)^11^. These medium contain 4.5 g/L glucose. Fresh medium exchange was performed every two days. To mature MoDCs, 100 ng/mL lipopolysaccharide binding protein (LBP) (Biometec GmbH, Germany) and 100 ng/mL LPS (Escherichia coli 026:B6 (SIGMA)) were added in each medium and the culture was continued for an additional 24 hours. The supernatants were collected and IL-12 (p40 + p70) was immediately determined by specific cytokine ELISA kits (Bender MedSystems) according to the manufacturer's instructions.

### MoDCs surface marker analysis

MoDCs were harvested and labeled with FITC-, PE- or APC-labeled mAbs (anti-human CD14, CD1c, CD40, CD80, CD83, CD86 HLA-DR, CCR7 or the relevant isotype controls: BD PharMingen, San Diego, CA), according to the manufacturer's directions. Briefly, the cells (0.5 × 10^5^) were incubated with 10 μL of Ab in a total volume of 50 μL (PBS) for 30 min at 4°C in the dark. Using a FACS Canto™ II (BD Immunocytometry Systems, San Diego, CA) flow cytometer, the cells were gated according to their size (forward light scatter: FSC), granularity (side light scatter: SSC), and surface marker expression and analyzed using the BD FACS Diva™ Ver6.1 (BD Immunocytometry Systems) program.

### Phagocytosis and Chemotaxis assay

Immature MoDCs that were generated with GM-CSF and IL-4 for 10 days in CCM were yielded and resuspended in 8 chamber polystyrene vessel tissue culture-treated glass slides (BD Falcon) with 400 μL CCM, HCM and ACM. To evaluate the endocytosis potential of DCs, in Fluoresbrite Plain Microspheres (2.5% Solids-Latex, 1.0 μm YG; Polysciences, Inc, Warrington, PA) was supplied to each medium. We confirmed that MoDCs phagocytosed microspheres by time-lapse fluorescence microscopy (Biozero BZ-8100; KEYENCE, Osaka, Japan). After 24 hr incubation, the DCs were washed and subjected to FACS analysis. Immature MoDCs were harvested and cultured under CCM, HCM and ACM with LPS for additional 24 hr. Chemotaxis of MoDCs was performed by μ-slide 6 (ibidi, Martinsried, Germany) as described in the manual. Briefly, a MoDCs suspension (15 μL) of 2.5 × 10^6^ cells/ml with each medium was seeded into the one chamber of a μ-slide 6 and incubated for 12 hr. One of the reservoirs was filled with the same medium with 2.5 nmol/mL CCL17. Cell movement was observed every 2 min for 120 min by time-lapse fluorescence microscope. Cell tracking and analysis were done using the manual tracking plug-in (Fabrice Cordelieres, Orsay, France) and the chemotaxis and migration tool (IBIDI) for Image J (U.S. National Institutes of Health, Bethesda, MD, USA), as described in the μ-slide chemotaxis protocol.

### Real-time PCR

CD14+ or CD1c+ cells were isolated from healthy volunteer and patients with advanced cirrhosis using magnetic microbeads. On real-time PCR, panels of 84 genes associated with mitochondrial energy metabolism and glucose metabolism were investigated simultaneously using the RT2 Profiler™ PCR A rrays (SAbiosciences, MD, USA). RNA samples were first reverse transcribed into cDNA templates by the RT2 First Strand Kit (SAbiosciences). The diluted cDNA templates were subsequently mixed with the RT2 qPCR Master Mix (SAbiosciences) and H_2_O. 25 μl of the mixture were loaded into each well of the array plates which contained pre-coated specific primers. The real-time PCR was performed as follows: an initial incubation at 95°C for 10 min, then 40 cycles at 95°C for 15 s and 60°C for 1 min. Data analysis was undertaken by using the SAbiosciences web-based PCR array data analysis software. On AMPK mRNA, after the extraction of total RNA and the RT procedure, real-time PCR using a TaqMan Chemistry System was carried out. The ready-made sets of primers and probes for the amplification of PRKAA1 (AMPK, Assay ID: Hs01562315_m1) was purchased from Perkin-Elmer/Applied Biosystems. The relative amount of target mRNA was obtained by using a comparative threshold cycle (CT) method.

### Measurement of ionic metabolite by using CE-TOFMS system

Immature MoDCs (1 × 10^6^ cells) were cultured from CD14+ cells of healthy volunteers under HCM and ACM. These media were removed from the plates, and 10 ml of 5% mannitol solution was added to wash the cells. After 2 ml of 5% mannitol solution was added to wash the cells, 1,300 μL of methanol containing 10 μM Internal Standard Solution 1 (Human Metabolome Technologies, Tsuruoka, Japan) were added. Cells were scraped from the plate and 1,000 μL of cell lysate were used. Then, 400 μL of Milli-Q water and 1,000 μL of chloroform were added to the samples, thoroughly mixed, and then centrifuged for 5 min at 2,300 g and 4°C. The 750 μL of the upper aqueous layer was centrifugally filtered through a Millipore 5-kDa cutoff filter to remove proteins. The filtrate was lyophilized and dissolved in 50 μL of Milli-Q water and analyzed by CE-TOFMS.

### CE-TOFMS Instrumentation

CE-TOFMS was carried out using an Agilent CE Capillary Electrophoresis System equipped with an Agilent 6210 Time of Flight mass spectrometer, Agilent 1100 isocratic HPLC pump, Agilent G1603A CE-MS adapter kit, and Agilent G1607A CE-ESI-MS sprayer kit (Agilent Technologies,Waldbronn, Germany). The system was controlled by Agilent G2201AA ChemStation software version B.03.01 for CE (Agilent Technologies, Waldbronn, Germany).

### CE-TOFMS conditions

Cationic metabolites were analyzed using a fused silica capillary (50 μm i.d. × 80 cm total length) with Cation Buffer Solution (Human Metabolome Technologies) as the electrolyte. The sample was injected at a pressure of 50 mbar for 10 sec (approximately 10 nl). The applied voltage was set at 27 kV. Electrospray ionization-mass spectrometry (ESI-MS) was conducted in the positive ion mode, and the capillary voltage was set at 4,000 V. The spectrometer was scanned from m/z 50 to 1,000. Other conditions were as in the cation analysis (Soga and Heiger 2000). Anionic metabolites were analyzed using a fused silica capillary (50 μm i.d. × 80 cm total length) with Anion Buffer Solution (Human Metabolome Technologies) as the electrolyte. The sample was injected at a pressure of 50 mbar for 25 sec (approximately 25 nl). The applied voltage was set at 30 kV. ESI-MS was conducted in the negative ion mode, and the capillary voltage was set at 3,500 V. The spectrometer was scanned from m/z 50 to 1,000. Other conditions were as in the anion analysis^35^.

### The Data analysis

Raw data obtained by CE-TOFMS were processed with MasterHands^36^. Signal peaks corresponding to isotopomers, adduct ions, and other product ions of known metabolites were excluded, and remaining peaks were annotated with putative metabolites from the HMT metabolite database (Human Metabolome Technologies Inc., Japan) based on their migration times (MTs) and m/z values determined by TOFMS. The tolerance range for the peak annotation was configured at ±0.5 min for MTs and ±10 ppm for m/z. In addition, peak areas were normalized against those of the internal standards and then the resultant relative area values were further normalized by sample amount.

### Immunoblotting

MoDCs were generated with GM-CSF and IL-4 under CCM for 10 days. MoDCs were harvested and cultured under CCM, HCM and ACM for an additional 48 hr. Each medium was removed and cells were lysesd using CelLyticTM-M Mammalian Cell Lysis/Extraction Reagent (SIGMA) with Protease Inhibitor Cocktail (SIGMA) and Phosphatase Inhibitor Cocktail 2 (SIGMA). The lysed cells were centrifugated for 10 minutes at 12,000–20,000 × g to pellet the cellular debris. Thereafter, the protein concentrations were determined by a Modified Lowry Protein Assay Kit (PIERCE, Rockford, IL). In total, 50 μg of protein were loaded onto NuPAGE 4–12% Bis-Tris gel (life technologies, Carlsbad, CA) and electro-transferred to a PVDF membrane (life technologies). After washing, the membranes were incubated in 25 ml of blocking buffer (LI-COR Biosciences, Lincoln, NE) for 1 hour at room temperature. Immunostaining was performed with the primary antibody (total AMPK, p-T172-AMPK, mTOR, phospho-p70 S6K; Cell Signaling Technology, Beverly, MA), followed by incubation with IRDye 800CW secondary antibody (LI-COR). Immunoreactive proteins were detected with an infrared imaging system (LI-COR). To confirm equal protein loading in all samples, the blot was stripped for 30 min in antibody stripping solution (Re-Blot Plus Western Blot Recycling Kit, Chemicon International, Temecula, CA), washed extensively and relabeled with anti β-actin antibody (SIGMA).

### Statistical analysis

The data were analyzed with ANOVA, and multiple comparisons were performed with Dunnett's post-hoc procedure. When 2 groups were analyzed, the differences between groups were analyzed by the Wilcoxon t-test. All data are expressed as mean SEM. In all analyses, a P value of less than 0.05 was considered statistically significant. All statistical analyses were performed with standard statistical software (JMP® Pro 9 for Windows).

## Author Contributions

E.K. designed research; E.K., Y.K., T.K., M.N. and O.K. performed research, E.K. analyzed the data; E.K., Y.U. and T.S. wrote manuscript; E.K., Y.K., T.K., M.N., O.K., Y.U. and T.S. reviewed the manuscript.

## Supplementary Material

Supplementary Information Supplemental information

Supplementary Information movie S1
