# Supplementary material for: Plasma amino acids imbalance in cirrhotic patients disturbs the tricarboxylic acid cycle of dendritic cell
Source: Sci Rep. 2013 Dec 10;3:3459. doi: 10.1038/srep03459 (PMC3857572; doi:10.1038/srep03459)
Supplement: Supplementary Information — Supplemental information [file srep03459-s1.doc]

Supplementary Information

Plasma amino acids imbalance in cirrhotic patients disturbs the tricarboxylic acid cycle of dendritic cell.

Eiji Kakazu, Yasuteru Kondo, Takayuki Kogure, Masashi Ninomiya, Osamu Kimura, Tatsuki Morosawa, Tomoaki Iwata, Yoshiyuki Ueno and Tooru Shimosegawa

Inventory of Supplementary Information

Supplementary Figures: Figure S1 – S4

Supplementary Tables: Table S1 - S3

Supplementary Movies: Movie S1

Supplementary figure

Figure S1. *The capacity of phagocytosis by MoDCs*


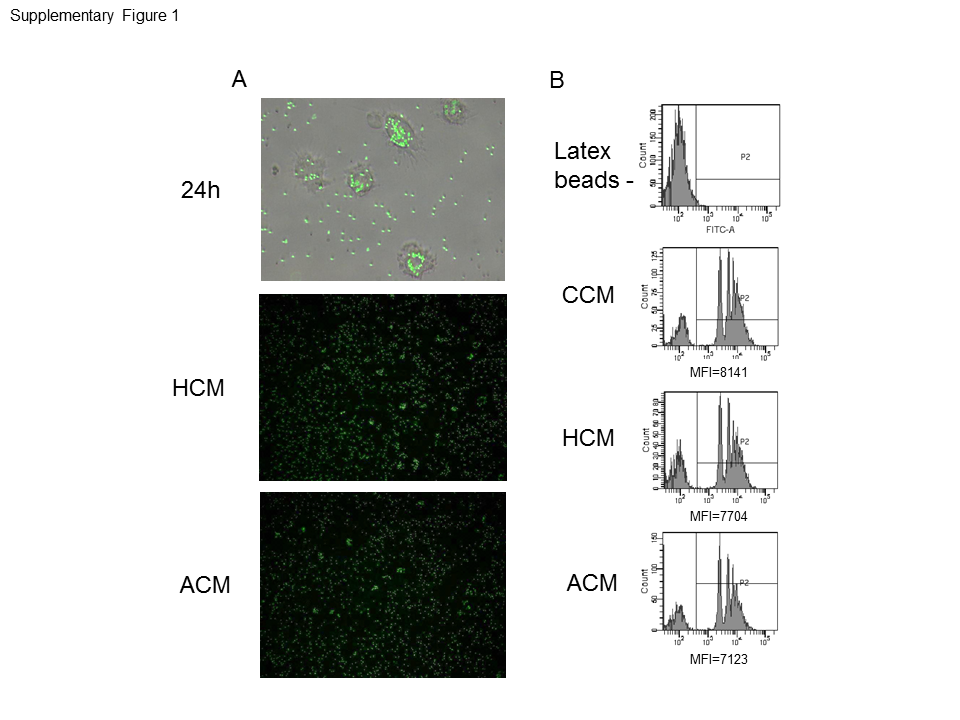
To evaluate the phagocytosis potential of immature MoDCs, Fluoresbrite Plain Microspheres were supplied to each medium for 24hr. (A) Fluorescence microscopy image. (B) The MoDCs were washed and analysed by flow cytometry. Results are representative of four experiments from four different donors.

Figure S2. *The consumption of amino acids by MoDCs under ACM and HCM*


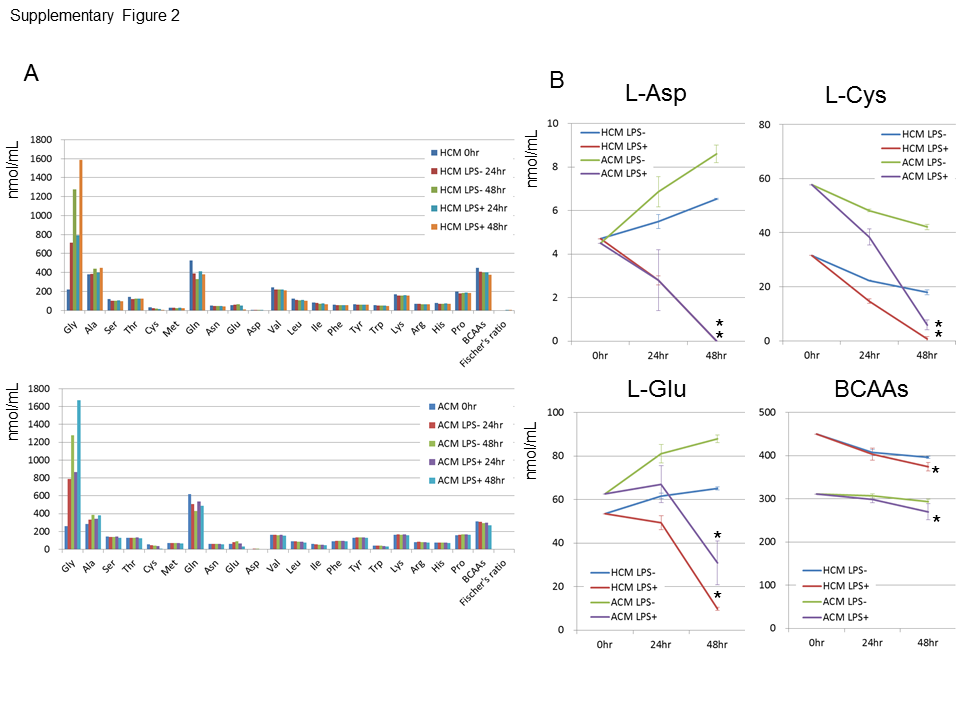


(A) Immature MoDCs were harvested and cultured under CCM, HCM and ACM with LPS for additional 48hr. The supernatants were collected at 24hr and 48hr, and the concentration of amino acids was measured by HPLC. (B) The consumption of L-Asp, L-Cys, L-Glu and BCAAs was significantly higher during maturation.

Figure S3. *Metabolomics of MoDCs cultured under ACM and HCM.*


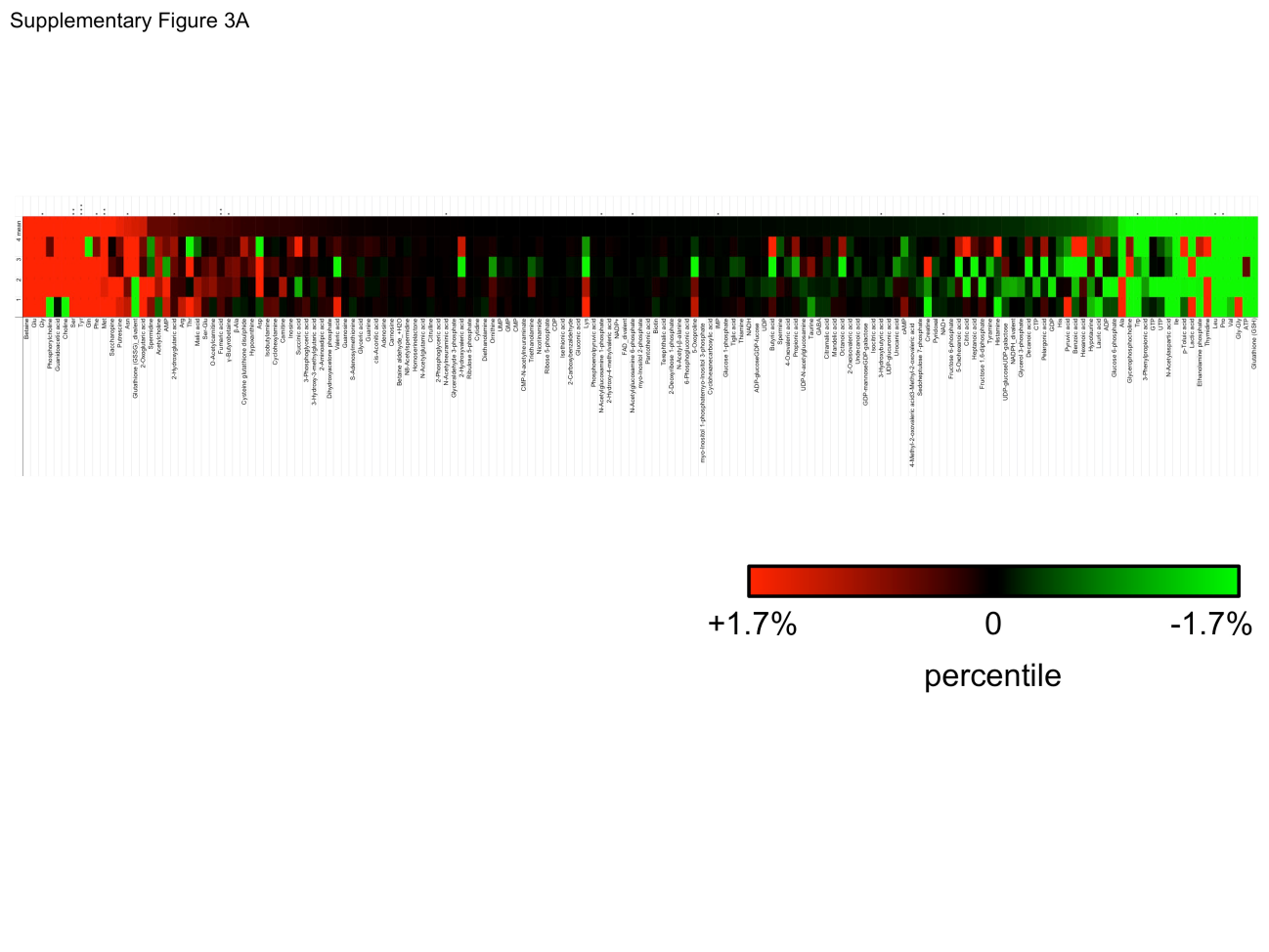

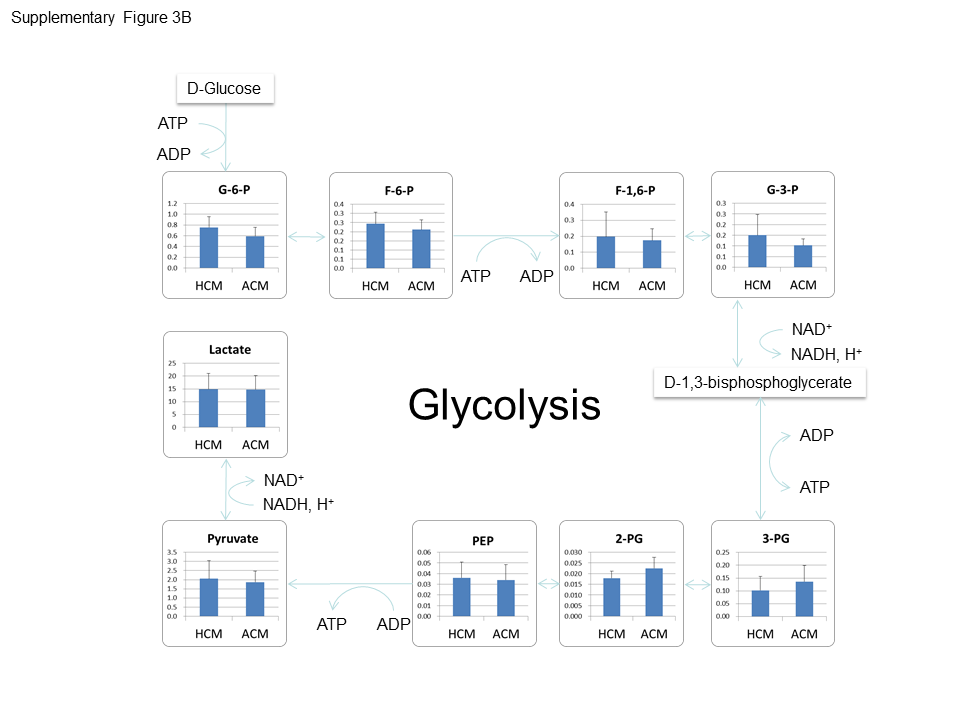


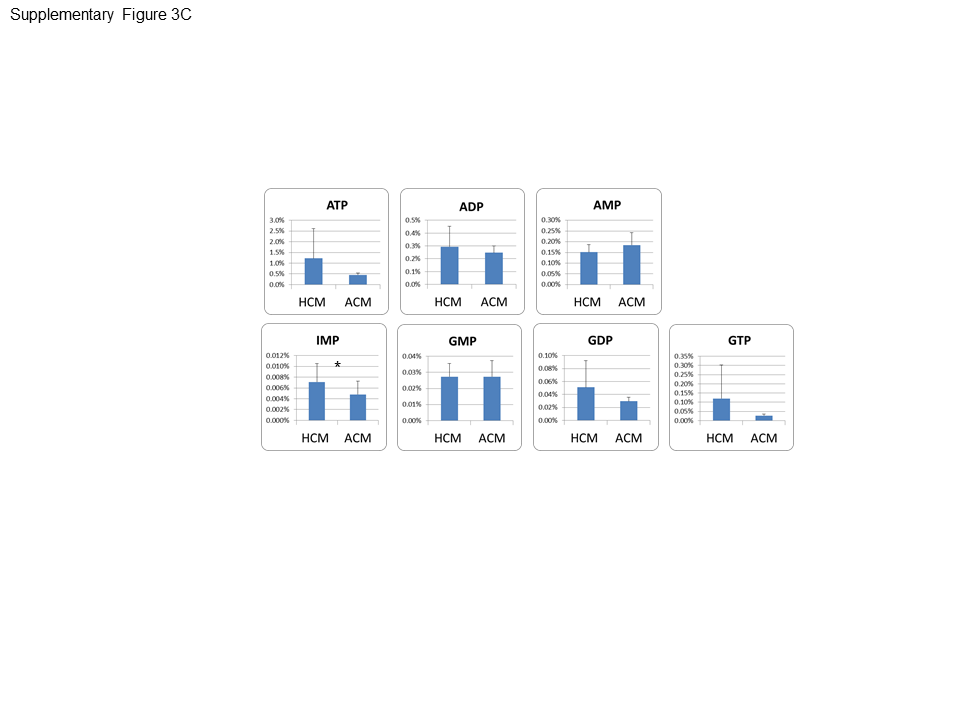


(A) We performed CE-TOFMS using extract of MoDCs cultured under HCM or ACM. We could analyse 159 of 163 metabolites. The differences in relative levels of MoDCs metabolites between HCM and ACM were visualized by using percentile heat map. Red tiles indicate increased metabolites and geen tiles indicate decreased metabolites under ACM. *** p<0.001, ** p<0.01, * p<0.05. (B) Metabolites in the glycolytic pathway. (C) purine metabolism.


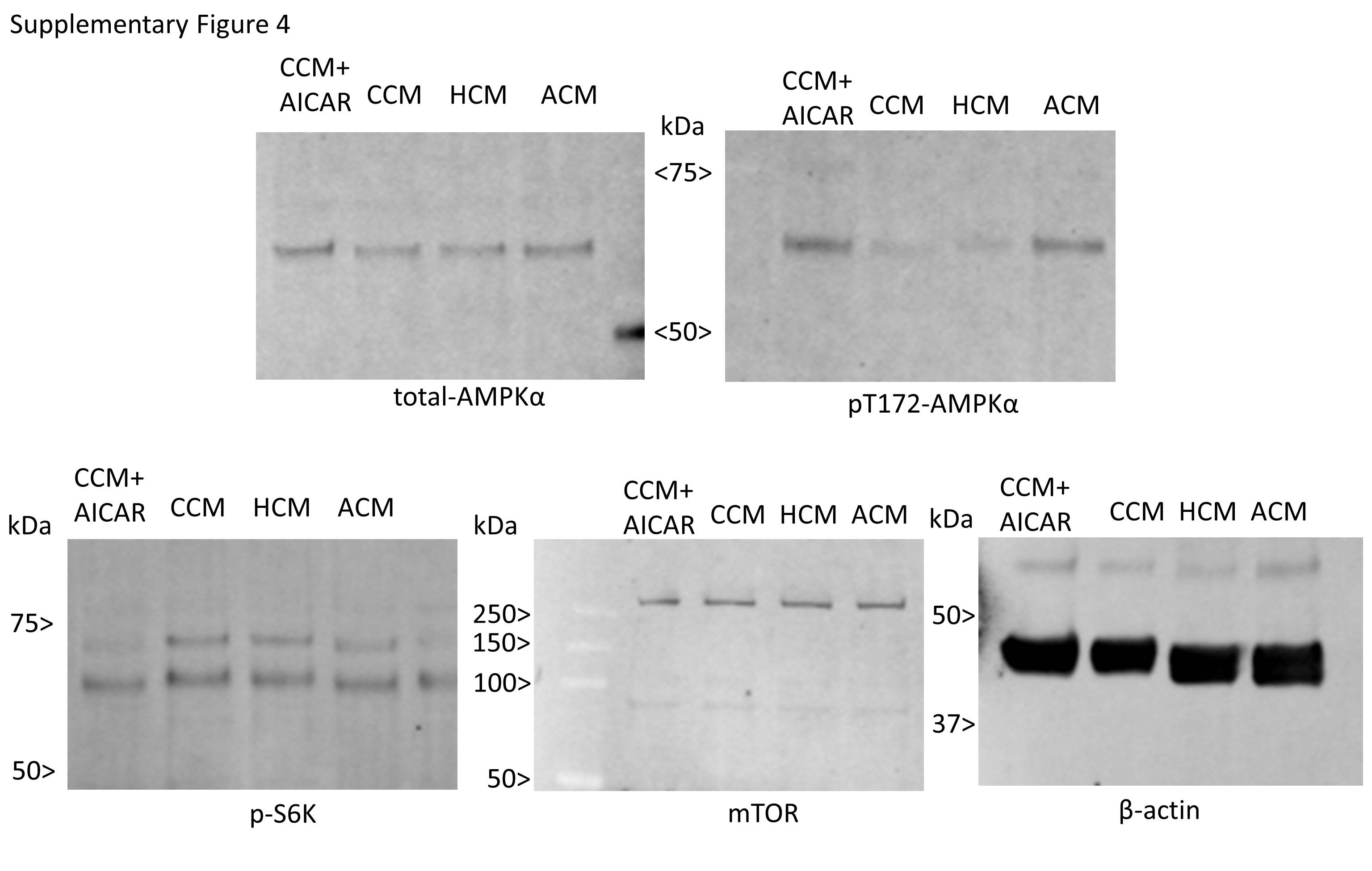


MoDCs were generated from CD14+ cells under CCM for 10 days and re-cultured under CCM, HCM and ACM for an additional 24 h. Cells were harvested and lysed. Equal amounts of protein were loaded and the levels of mTOR, phosho-p70 S6K, phosphor-T172 AMPK and total AMPK were determined by Western blot analysis.

Supplemental table

Table S1. An imbalance in the plasma amino acids appeared in accord with the progression of cirrhosis.

|  |  | Child-Pugh grade | |
| --- | --- | --- | --- |
|  | CH (n=107) | A (n=165) | B and C (n=105) |
| Glycine | 221 | 217 | **260** |
| L-Alanine | 375 | **320** | **323** |
| L-Serine | 126 | 133 | **149** |
| L-Threonine | 136 | 147 | **153** |
| L-Cystine | 48 | 52 | **66** |
| L-Methionine | 31 | 35 | **62** |
| L-Glutamine | 590 | 620 | **677** |
| L-Asparagine | 53 | 56 | **68** |
| L-Glutamic acid | 72 | **59** | **52** |
| L-Aspartic acid | 4.0 | 3.2 | 3.8 |
| L-Valine | 238 | **213** | **186** |
| L-Leucine | 132 | **116** | **104** |
| L-Isoleucine | 68 | **61** | **56** |
| L-Phenylalanine | 73 | 79 | **100** |
| L-Tyrosine | 87 | **97** | **135** |
| L-Tryptophan | 54 | 52 | **45** |
| L-Lysine | 205 | 194 | 188 |
| L-Arginine | 71 | 80 | **88** |
| L-Histidine | 86 | 84 | 84 |
| L-Proline | 162 | 157 | 176 |
| Fischer's ratio | 2.81 | **2.31** | **1.55** |

The concentrations of the plasma amino acids from fasting chronic hepatitis (n=107), patients with early cirrhosis (n=165) and patients with advanced cirrhosis (n=105) were measured by HPLC in the early morning. CH: chronic hepatitis. Thick font indicates significant change vs CH (p<0.01) by Dunnett’s test.

Table S2. *Characteristics of study participants*

LC-C: liver cirrhosis due to HCV

PBC: Primary biliary cirrhosis

AST / ALT (IU/L)

Total Bilirubin (mg/dL)

Albumin (g/dL)

PLT: platelet counts (x10^3^/μL)

PT-INR: prothrombin time-international normalized ratio

Fischer’s ratio mean: Valine+Leucine+Isoleucine / Tyrosine+Phenylalanine

The MELD score [^1^](#_ENREF_1) was calculated by an on-line worksheet available on the internet at www.mayoclinic.org/meld/mayomodel5.html.

*Table S3. The serum free culture media used in this study (nmol/mL).*

|  | CCM | HCM | ACM |
| --- | --- | --- | --- |
| Glycine | 400 | 225 | 280 |
| L-Alanine | 400 | 391 | 307 |
| L-Serine | 400 | 119 | 151 |
| L-Threonine | 800 | 142 | 138 |
| L-Cystine 2HCl | 200 | 38 | 67 |
| L-Methionine | 200 | 29 | 75 |
| L-Glutamine | 4000 | 564 | 689 |
| L-Asparagine | 400 | 51 | 64 |
| L-Glutamic Acid | 400 | 42 | 53 |
| L-Aspartic Acid | 400 | 3 | 4 |
| L-Valine | 800 | 249 | 175 |
| L-Leucine | 800 | 132 | 100 |
| L-Isoleucine | 800 | 76 | 53 |
| L-Phenylalanine | 400 | 63 | 99 |
| L-Tyrosine | 400 | 65 | 133 |
| L-Tryptophan | 80 | 62 | 45 |
| L-Lysine-HCl | 800 | 183 | 184 |
| L-Arginine-HCl | 400 | 78 | 92 |
| L-Histidine HCl-H2O | 200 | 83 | 85 |
| L-Proline | 400 | 204 | 176 |
| Fischer's rate | 3.00 | 3.57 | 1.42 |

Complete culture medium (CCM)’ containing 20 amino acids that are relevant to the make-up of mammalian proteins. HCM (healthy control medium): consistent with the average concentration of plasma amino acids from healthy volunteers (n=25). ACM (advanced cirrhotic medium): consistent with the average concentration of plasma amino acids from patients (Child-Pugh grade B or C, n=43). Components other than amino acid were identical among media. We verified that there was no difference between the theoretical value and actual value by HPLC. The amino acid concentrations are expressed in nmol/mL. Fischer’s ratio mean : Valine+Leucine+Isoleucine / Tyrosine+Phenylalanine.

Movies S1. *MoDCs Phagocytose Microspheres*

MoDC phagocytosis was observed every 1 min for 20 min by time-lapse fluorescence microscope. Green spots are Microspheres and orange fluorescence is JC-1 dye.

Supplementary Experimental Procedures

Reference

1. Kamath PS, Wiesner RH, Malinchoc M, Kremers W, Therneau TM, Kosberg CL*, et al.* A model to predict survival in patients with end-stage liver disease. *Hepatology (Baltimore, Md)* 2001, **33**(2)**:** 464-470.
